# Remarks on the Treatment of Gunshot Wounds of the Abdomen in Relation to Modern Peritoneal Surgery

**Published:** 1882-07

**Authors:** J. Marion Sims


### REMARKS ON THE TREATMENT OF GUNSHOT WOUNDS OF THE ABDOMEN IN RELATION TO MODERN PERITONEAL SURGERY. By J. MARION SIMS, M. D., LL.D., etc.

Occasionally wounds of the abdomen above the brim of the pelvis recover; but it is always when there is a chance for drainage. Dr. Pallen saw a Confederate soldier recover, where a large part of the abdominal parietes were blown off by a bursting shell. The wound was immense, and diagonally across the abdomen. The bowels fell on the ground, but were not wounded.

They were washed clean, returned to their proper place, and the wound was closed as well as possible with strong coarse thread, leaving an open space six inches long and two inches wide, through which the intestines could be seen. The were retained *in situ* by compress and bandage, and the man eventually recovered without a single drawback. He recovered because the intestines were not injured, and because there was drainage. This was a case in which we might have supposed there was danger of peritonitis; but peritonitis and free drainage are antagonistic.

Dr. Newall, of New Brunswick, New Jersey, reports to me the following interesting case: A large man, with a "full abdomen," shot himself accidentally with a shot-gun in 1847. The charge entered the abdomen an inch below the sternum. and to the left of the median line. Introducing the finger, Dr. Newell plainly felt the left lobe of the liver and the inner wall of the abdomen. The next day twenty-nine shot passed the bowels. Three shot made their exit one inch and a half above the crest of the ilium, and three and a half inches from the spine. "An incision was made here into the cavity of the abdomen, and I removed the gun-wad, composed of tow, paper, shot, a printer's type and a piece of the man's vest—in all, sufficient to fill a tumbler half-full. The patient was kept under the influence of opium for several days. He recovered completely, and is now living." In this case Dr. Newall did, thirty-four years ago, what I now advise to be done in all cases of gun-shot wounds of the abdominal cavity. His patient was saved by the removal of foreign bodies, and by an opening in the iliac region, which permitted free drainage.

Having now brought forward all the facts observed by myself and by personal friends, let us turn to the records of the American Civil War for further facts illustrating this important question.

1. *Wounds of the Stomach.*—There were sixty-four shot wounds of the stomach, more or less complicated with wounds of neighboring parts. There was but one well authenticated case of recovery. Other reported cases of recovery were carefully analyzed by Otis, and pronounced wholly unreliable.

There were nine cases of bayonet wounds penetrating the peritoneal cavity without lesion of viscera. Six terminated favorably. Seven had traumatic peritonitis. The diagnoses were not clear in every case.

There were four fatal punctured or incised wounds of the stomach. Five fatal shot wounds are reported as having survived seven, eight and nine days, and one forty days. The length of time that these survived indicates clearly how different the result might have been in some of them, if prompt measures had been taken for exploring the seat and nature of the wound, and resorting to gastrorrhaphy, the only rational treatment in such cases. There was not a single case of gastrorraphy during the whole war. This is explained by the fact that the stomach does not prolapse through an ordinary bullet wound; and surgeons did not then dare to enlarge such wounds to investigate the nature and extent of internal injuries.

There were three cases of secondary fistulæ, which terminated fatally. One lived four weeks, another seven weeks, and the third eighty days. Timely interference and gastrorrhaphy would almost certainly have saved all of them.

Considering the facility and comparative safety with which gastric fistulæ are now established by surgical means, it is a little surprising that (according to Otis) we have the record of but two cases which have survived shot wounds of the stomach for any length of time. The first "is that of Maillot, wounded in Möllendorf's repulse of the French at Kaiserslautern, in May, 1794. In this little known, but authentic case, recorded by Baron Percy, the fistulæ gradually contracted, and ultimately closed." The other is the well known case of Alexis St. Martin, made famous by Beaumont, who, nearly fifty years ago (1833) published an account of the digestibility of various article of diet as observed in the stomach of St. Martin. Beaumont states that St. Martin, a Canadian, of French parentage, was about eighteen years old when wounded (June 6, 1822) at Fort Mackinau, where Dr. Beaumont was stationed as surgeon to the post. As St. Martin died only a few months ago, he survived injury about fifty-nine years.

Dr. Otis sums up wounds of the stomach in the following words: There were "four fatal punctured or incised wounds; one incontestable recovery from a shot perforation; a few recoveries from shot wounds in the gastric region, in which the diagnoses were not determined unequivocally; and nearly sixty fatal cases of more or less complicated shot-wounds of the stomach."

The records of military surgery (according to Otis), from its earliest period to the present time, furnish but six or seven well-authenticated cases of recovery from shot-wounds of the stomach, with or without fistula. To this list must now be added another example of recovery from undoubted shot-wound of the stomach. It is the case of the distinguished gynæcologist, Dr. R. Beverly Cole, of San Francisco. I have just received a letter from him, dated London, January 17, 1882, detailing the following particulars: Dr. Beverly Cole, at the age of twenty five, resided in San Francisco, where he had suffered from repeated attacks of intermittent fever. When just recovering from one of these, he left his house, on June 3, 1854, without taking breakfast; his stomach was therefore empty. Whilst in the act of packing his trunk, preparatory to making a visit to the country, a Colt's six-inch revolver (old pattern) fell from his inside breast coat pocket, the body being bent over the trunk at the time, and the hammer of the pistol striking the edge of the trunk as it fell, the cap was exploded, and the ball entered the breast—the muzzle not being more than eight inches from the body. He did not fall, but, raising himself up, he tore open his vest and shirt, and saw that he was wounded. Syncope occurring, a friend caught and laid him on a sofa near by. When consciousness returned, he found himself surrounded by a number of his medical friends, among whom were Drs. C. S. Tripler and H. S. Hewitt, of the United States army, and Drs. Valentine Mott, Jr., A. B. Stout and Charles Bertody. He was totally blind, but recognized them all by their voices. He heard Dr. Tripler say: "Never mind the ball; it can be sought for at any future time. We must first bring about reaction." Soon after this he was suddenly seized with an indescribable pressure in the rectum, and a desire to defæcate. Morphia was administered, sinapisms were applied to the extremities, and ammonia was given in very minute quantities—minute, for fear of its escaping through the gastric wound into the peritoneal cavity. As reaction came on, the sensation in the rectum increased till he vomited nearly a wash-hand bowlful of blood, black and partially coagulated. It was estimated by the attending physicians to be from a quart to a half-gallon, or more. This gave some relief. But the rectal pain and tenesmus were not completely relieved till he was brought fully under the influence of morphia. As he lay on his back, his clothing was all cut away without turning him on either side, and he was then placed in bed. The collapse was very complete, and several hours elapsed before reaction was fully established. During all this time he could not see; but from the conversation of the surgeons and from the frequency with which they examined the cardiac region, he inferred that death was imminent. The sinapisms were forgotten, and were not removed for four or five hours, and they produced sloughing ulcers, which were nearly twelve months in healing. When reaction was fully established, Dr. Tripler passed the end of the little finger along the track of the ball, through the conjoined cartilages of the seventh and eighth ribs—an inch and a half to the left of the median line of the ensiform cartilage. He then passed a probe along it into the stomach. The lodgment of the ball was not discovered for two weeks or more later. It was then found between the eleventh and twelfth ribs on the back, two inches to the left of the median line. This showed that the course of the ball was directly through the body—the difference between the parallels of entrance and exit being due to the difference between the bent and the erect posture.

For three weeks he was nourished by the rectum. Beef-tea was thus given every three hours; at first one ounce, then two, then three, and finally four ounces. During this time a small quantity of beef tea was given by the mouth, but it produced such severe pain, as it entered the stomach, that it was not soon repeated. Small lumps of ice were allowed to quench the thirst produced by the morphia, which was given in half-grain doses three or four times a day, or whenever needed. On the twelfth day the bowels were moved by enema. On the twenty-first day he was removed to his own home. He then began to suffer from severe paroxysms of pain in the back, which were so intense as to obstruct respiration. They continued without abatement for three weeks. Dr. Tripler then removed the ball, and they ceased. He was confined to bed six weeks. When he got up it was discovered that the left shoulder was lower than the right, the result of a constrained position while in bed; and there was a dragging sensation in the gastric region, not only disagreeable, but quite painful, as if the stomach had formed unnatural adhesions. On account of these disabilities, he was compelled to go on crutches for two years before his body attained its natural erect manner of carriage.

The posterior wound closed in a few days after the removal of the ball; but the anterior wound did not close for four years, which was doubtless due to the injury of the cartilages, which are always tardy in reparation. For many years an ordinarily hearty meal (in consequence of adhesions between the stomach and contiguous parts) produced a dragging, uneasy sensation, which rendered life very uncomfortable.

Recovery was eventually complete; and no one now would suspect that he had ever been the subject of such a serious accident. A peculiar feature of the case was total loss of vision for three days, during which time he could not distinguish daylight. There can be no doubt that the ball in this case perforated the stomach. The large quantity of blood vomited soon after the wounding establishes the diagnosis beyond question. From the point of entrance and direction of the ball, it must have passed through the stomach, below the lesser curvature. As the ball was very small, the wound of the stomach was likewise very small; hence there was less probability of gastric effusion than if the ball had been larger. But recovery was chiefly due to the fact that the stomach was quite empty at the time of the accident. If it had been even partially full there would have been effusion into the peritoneal cavity, followed by certain death.

The history of Dr. Beverly Cole's case was published in the *Detroit Medical Journal* in 1855 or 1856, by Dr. C. S. Tripler, United States Army. But as Dr. Otis insinuated, in a note to the *Surgical History of the War* (Part II., "Surgery"), that the case was not incontestably one of the stomach, I place it on record here that others may judge for themselves.

2. *Wounds of Small Intestines.*—Of about six hundred and fifty cases of penetrating wounds of the abdomen during the war, fifty were of the small intestines, eighty-nine of the large intestines, and over five hundred in which the location of the wound was not discriminated, or was complicated with other lesions. Very few sword or bayonet wounds of the abdomen came under treatment, but a number of such injuries were observed among the slain on the field of battle. Wounds of the small intestines are more frequent than those of other portions of the alimentary canal, and are attended with higher mortality. They are more exposed to injury, being less protected by bony structure than other viscera. The ileum is most exposed, jejunum less, and the duodenum still less. Wounds of this portion of the canal are regarded as almost necessarily fatal. Of shot wounds of the small intestines during the war, Otis says: "It may still be doubted if an incontestable instance of recovery was observed." There were five cases of wounds of the duodenum, all complicated with wounds of other organs. They all died. One survived eight days; another twenty-four days. If the external wound had been sufficiently enlarged in time, the wounded portions might have been exsected, and the ends sutured, with chance of recovery.

3. *Wounds of Large Intestines.*—Injuries of this group are less fatal than wounds of small intestines. There were a few instances of recovery from shot wounds of the transverse colon; many after perforation of the cæcum and ascending portion of the bowel, and a still larger number in wounds of the sigmoid flexure and contiguous part of the descending colon. Many of these were complicated with injuries of the innominata. Nearly all resulted in fæcal fistulæ, which usually closed after a while without surgical interference. There were, in all, fifty-nine recoveries out of eighty-nine shot wounds of the cæcal and sigmoid extremities of the large intestine. Fæcal fistulæ persisted in nine, and were closed in fifty; seventeen within a month, twenty-eight within a year, and five at periods from one to five years. Ten per cent. of all slain in battle die of wounds of the abdomen, and from three to four per cent. of the wounded who come under treatment are wounded in the abdomen.

4. *Wounds of the Bladder.*—Baron Larrey was the first to show that gun-shot wounds of the bladder were less dangerous than those made by puncture or incision; because the tissues are so crushed by the missile that eschars are produced, which protect the connective tissues from urinary infiltration. There was no case of punctured, incised or lacerated wound of the bladder in our war. Of one hundred and eighty-three shot wounds of the bladder, eighty-seven (47.5 per cent.) survived. A large majority of these suffered from grave disabilities, and many from distressing infirmities, a few of them dying after years of suffering; several recovered, with permanent urinary fistulæ. Some had recto-vesical fistulæ, which closed early in a few, and later in others. However, it is rare to find the functions of the bladder perfectly restored after shot injuries. Shot wounds of the bladder are often complicated by the presence of foreign substances in it—such as bullet, bone or other material—which serve as nuclei for calculous deposits. There were twenty-one cystotomies for the removal of such formations. Many cases died from infiltration of urine into the cellular tissue, and many from extravasation of urine into the peritoneal cavity. Cystorraphy, recommended by Legouest, was not practiced.

The teachings of Vincent, of Lyons, and of Fischer, of Buda-Pesth, at the late International Medical Congress, alluded to in early part of this paper, prove that we may now safely undertake cystorrhaphy in all wounds of the bladder. Abdominal section, and cystorrhaphy, and clearing out thoroughly the peritoneal cavity, are the only means of safety in shot or other wounds of the bladder, where there is urinary extravasation into the peritoneum.

5. *Shot Wounds of the Rectum.*—One hundred and three shot-wounds of the rectum were reported, of which forty-four (or 42.7 per cent.) resulted fatally. Thirty-four of the cases, of which four were fatal, were complicated with wounds of the bladder. Fæcal fistulæ, after shot perforation of the rectum, were not uncommon, and were more persistent than cæcal or sigmoid fistulæ. Shot wounds of the rectum are not so dangerous as those of the upper bowels, but are about on a par with wounds of the cæcal and sigmoid ends of the colon. Hæmorrhage was not a frequent complication of shot-wounds of the rectum.—*British Medical Journal.*

